# Macrophage Activation Syndrome as an Atypical Manifestation of Mixed Connective Tissue Disease in a 14-Year-Old Girl: A Case Report

**DOI:** 10.7759/cureus.80206

**Published:** 2025-03-07

**Authors:** Hironori Yamaoka, Ichiro Hada, Masae Watanabe, Ryota Kurayama, Kunimasa Yan

**Affiliations:** 1 Department of Pediatrics, Kyorin University Suginami Hospital, Tokyo, JPN; 2 Department of Pediatrics, Kyorin University School of Medicine, Tokyo, JPN

**Keywords:** autoimmune disorder, hemophagocytic lymphohistiocytosis syndrome, hemophagocytic syndrome (hs), macrophage activation syndrome (mas), mixed connective tissue disease, rheumatic disorder, systemic juvenile idiopathic arthritis, systemic lupus erythematosus

## Abstract

Macrophage activation syndrome (MAS) is a subtype of secondary hemophagocytic lymphohistiocytosis (HLH) associated with rheumatic diseases. It is a life-threatening condition characterized by hypercytokinemia due to uncontrolled T-cell and macrophage activation. MAS is an uncommon complication in patients with mixed connective tissue disease (MCTD), particularly in those without a prior diagnosis or treatment. We report the case of a previously healthy 14-year-old Japanese girl admitted to our hospital with severe headache and persistent fever. Despite an initially unremarkable examination, she subsequently developed altered mental status, arthralgia, and a small erythema. Laboratory findings revealed cytopenia and coagulation abnormalities, along with elevated levels of C-reactive protein, liver enzymes, triglycerides, and ferritin. Although she did not meet the HLH-2004 diagnostic criteria established by the Histiocyte Society, MAS was suspected based on early diagnostic criteria for MAS associated with other rheumatic diseases. Prompt initiation of steroid pulse therapy led to rapid clinical improvement. Further serological testing revealed strongly positive anti-U1-ribonucleoprotein (RNP) antibodies, confirming a diagnosis of MCTD. Over one year of follow-up, the patient remained well-controlled on a tapered dose of prednisolone, with no serological relapse. This case highlights the importance of early diagnosis and treatment of MAS, even when the underlying disease is unknown. Additionally, cytokine profiling may provide valuable insights into understanding the pathogenesis of diseases that cause hypercytokinemia.

## Introduction

Macrophage activation syndrome (MAS) is a subtype of secondary hemophagocytic lymphohistiocytosis (HLH) associated with rheumatic diseases. It is a severe condition characterized by fever, pancytopenia, and hepatosplenomegaly that rapidly progresses to disseminated intravascular coagulation and multiorgan failure [1]. Systemic-onset juvenile idiopathic arthritis (sJIA), systemic lupus erythematosus (SLE), and adult Still’s disease (ASD) are common underlying diseases, whereas mixed connective tissue disease (MCTD) is rare [2]. There have been some reports of MAS developing during treatment for MCTD [2-3]; however, MAS rarely develops in untreated or undiagnosed patients. Here, we report a case of a previously healthy adolescent girl who developed MAS and was eventually diagnosed with MCTD.

This case was previously presented as a meeting abstract at the 128th Annual Meeting of the Japan Pediatric Society on April 18 to 20, 2025.

## Case presentation

A 14-year-old Japanese adolescent girl presented to our emergency department with a severe headache and a 5-day history of fever. She had experienced occasional Raynaud’s phenomenon and hand swelling for the past two years before visiting our hospital; however, she had not sought medical attention. She had no other medical or family history of rheumatic or hematological diseases and was not on any medications. On physical examination, the patient had clear lung sounds bilaterally, a regular heart rhythm without murmurs, normal bowel sounds with a soft abdomen, no swelling of the extremities, and no skin rash. There were no neuropsychiatric signs, including meningeal signs. Initial blood test results (Table 1) revealed no significant findings, except for slightly elevated C-reactive protein (CRP; 1.71 mg/dL) levels and hyponatremia (128 millimoles/L). She was admitted to our pediatric department for severe headache and persistent fever.

**Table 1:** Laboratory investigations WBC: white blood cells; PLT: platelets; HGB: hemoglobin; AST: aspartate aminotransferase; ALT: alanine aminotransferase; LDH: lactate dehydrogenase; CK: creatine kinase; BUN: blood urea nitrogen; CRP: C-reactive protein; sIL-2R: soluble interleukin-2 receptor; PT-INR: prothrombin time international normalized ratio; APTT: activated partial thromboplastin time; IL-6: interleukin-6; IL-18: interleukin-18; CXCL9: C-X-C motif chemokine ligand 9; sTNF-RII: soluble tumor necrosis factor-receptor II; IFN-α: interferon-α

	Day 1 (On admission)	Day 4 (At the onset of MAS)	Day 44 (At follow-up)	Reference range
WBC (×10^3^/μL)	3.9	1.8	10.1	4.0-10.5
PLT (×10^3^ /μL)	190	127	307	150-400
HGB (g/dL)	14.7	12.9	14.0	12.0-15.0
AST (U/L)	34	77	15	10-40
ALT (U/L)	15	30	17	5-45
LDH (U/L)	390	887	239	120-330
CK (U/L)	62	163	25	5-130
BUN (mg/dL)	14.6	6.5	11.3	7-18
Creatinine (mg/dL)	0.66	0.48	0.42	0.31-0.88
CRP (mg/dL)	1.71	7.99	<0.10	0.06-0.81
Triglycerides (mg/dL)	118	146	123	<140
Ferritin (ng/mL)	266.9	1518.9	62.2	10-70
sIL-2R (U/mL)	—	1980	—	122-496
Sodium (mmol/L)	128	131	141	135-145
Potassium (mmol/L)	4.6	3.6	4.2	3.3-4.6
Chloride (mmol/L)	95	97	102	98-108
PT-INR	1.18	1.29	0.91	1.0
APTT (s)	38.1	38	29.5	30-40
D-dimer (μg/mL)	4.3	88.5	<0.5	<1.0
Fibrinogen (mg/dL)	—	343	251	177-420
IL-6 (pg/mL)	—	63	<3	<3
IL-18 (pg/mL)	—	1145	636	<500
CXCL9 (pg/mL)	—	14502	31	<31-83
sTNF-RII (pg/mL)	—	13893	2718	<829-2262
IFN-α (pg/mL)	—	27	5	<3

Following admission, her fever persisted, while her headache resolved spontaneously. On the fourth day of hospitalization, she suddenly developed altered mental status. Although she remained conversant, she exhibited slowed verbal responses and disorientation to time. By the time we examined her, her mental status had returned to normal, and she showed no neuropsychiatric symptoms such as delirium, psychosis, or seizures. Although her altered mental status was transient, she developed arthralgia in her left elbow and a small erythema on her chest. Laboratory tests (Table 1) revealed cytopenia (white blood cells 1.8×10^3^ /µL, platelets 127×10^3^ /µL, hemoglobin 12.9 g/dL), elevated levels of CRP (7.99 mg/dL) and liver enzymes (aspartate aminotransferase 77 U/L, alanine aminotransferase 30 U/L, lactate dehydrogenase 887 U/L), and coagulation abnormalities (fibrinogen 343 mg/dL, D-dimer 88.5 μg/mL). Furthermore, elevated levels of triglycerides (146 mg/dL), ferritin (1518.9 ng/mL), and soluble interleukin-2 receptor (sIL-2R; 1980 U/mL) were also noted. A peripheral blood smear showed a left shift in neutrophils without hemophagocytosis. Complement activity was within normal physiological ranges (C3 141 mg/dL, C4 32 mg/dL, 50% hemolytic complement activity 41.3 U/mL), and serology was negative for Cytomegalovirus, Epstein-Barr virus, and Herpes simplex virus. To identify the source of her prolonged fever, we performed cardiac and abdominal ultrasonography, along with computed tomography (CT) of the torso. We also performed brain magnetic resonance imaging (MRI) to assess for intracranial disease. Imaging studies revealed no significant abnormalities. A lumbar puncture was not performed, as meningitis or encephalopathy was not suspected given her clear mental status and lack of neurological findings. MAS was suspected based on her history and symptoms related to rheumatic diseases, and the laboratory findings suggestive of hypercytokinemia. Steroid pulse therapy with methylprednisolone (1000 mg/day for three days per course) was initiated on the same day.

The following day, the patient’s symptoms and laboratory results showed a marked improvement. Furthermore, her blood tests revealed strongly positive anti-U1-ribonucleoprotein (RNP) antibodies (> 550 U/mL) and weakly positive anti-Smith (Sm) antibodies (12.7 U/mL) and rheumatoid factor (21.4 U/mL). Anti-double-stranded deoxyribonucleic acid (dsDNA), anti-SS-A, anti-SS-B, anti-cardiolipin, anti-Scl-70, anti-Jo-1, anti-cyclic citrullinated peptide (CCP), and anti-neutrophil cytoplasmic antibodies were all negative. Renal histology was normal, with no findings suggestive of lupus nephritis. She met the Japanese diagnostic criteria for pediatric MCTD; thus, we concluded that she had developed MAS as a manifestation of MCTD. Two courses of intravenous steroid pulse therapy were administered with a one-week interval between courses. Prednisolone (60 mg/day) was then initiated as post-treatment. After discharge, we gradually tapered her prednisolone dosage from 30 to 3 mg/day over one year. Her condition remained well-controlled with no serological relapse, despite experiencing episodic Raynaud’s phenomenon and hand swelling.

## Discussion

This report highlights a rare case of MAS as an atypical manifestation of MCTD in a pediatric patient. MAS is a subtype of secondary HLH caused by rheumatic diseases. HLH is a lethal condition caused by severe hypercytokinemia due to an uncontrolled and sustained activation of the immune system, including cytotoxic T cells and macrophages [1]. Histologically, HLH is characterized by macrophage proliferation and a hemophagocytosis picture in the bone marrow and other reticuloendothelial tissues. It can be categorized into the following two types according to its cause: primary HLH due to genetic abnormalities and secondary HLH due to infections, rheumatic diseases, or malignancies. Ishii et al. in Japan reported that infections were the most common cause of HLH (64%), followed by malignancies (18%), rheumatic diseases (11%), and primary HLH (2%) [4]. Furthermore, a systematic review by Atteritano et al., on 421 patients with MAS, reported that the underlying rheumatic diseases included sJIA, SLE, ASD, rheumatoid arthritis, dermatomyositis, and systemic scleroderma in 219/421 (52%), 94/421 (22.3%), 37/421 (8.8%), 13/421 (3.1%), 7/421 (1.7%), and 5/421 (1.1%) patients, respectively [2]. Only one case of MCTD in an adult patient was noted in this review. Our review of the existing literature indicated reports of MAS developing during the course of treatment for MCTD [2,3], and one report of MAS in an adult patient without a previous diagnosis of MCTD [5]; however, no pediatric cases have been reported to date. In our case, we diagnosed the patient with MAS caused by MCTD. There were few findings suggesting infectious diseases, as she had no system-specific symptoms (e.g., respiratory or gastrointestinal symptoms), and all tests for infection, including blood bacterial culture and viral-specific antibody or genetic testing, were negative. The imaging studies did not reveal the source of her prolonged fever or lesions suggestive of malignancies. The patient’s history of Raynaud’s phenomenon and hand swelling, even before the onset of her current symptoms, suggested an underlying autoimmune condition. Combined with the presence of positive anti-U1-RNP antibodies, we made a diagnosis of MCTD. Additionally, the rapid improvement in both clinical symptoms and laboratory findings after steroid pulse therapy further supports our diagnosis.

Due to high mortality rates in the initial stages of disease onset, early diagnosis and treatment of MAS are essential even when the underlying disease is unclear [2,6]. The HLH diagnostic criteria, revised by the Histiocyte Society in 2004 (HLH-2004 criteria) (Table 2) are used worldwide for diagnosing HLH [7]. A clinical diagnosis can be made in a patient who meets five of eight of the following criteria: fever, splenomegaly, bicytopenia, hypertriglyceridemia, or hypofibrinogenemia, pathology confirming hemophagocytosis, low natural killer (NK) cell activity, elevated ferritin counts, and high sIL-2R levels. However, the HLH-2004 criteria were primarily developed for diagnosing primary HLH patients. Patients with secondary HLH often do not meet these criteria until the disease has progressed to a severe condition, causing multiorgan failure, and there are no clinically useful early diagnostic indicators. Therefore, early diagnostic criteria for MAS associated with sJIA (sJIA-MAS) [8] and MAS associated with SLE (SLE-MAS) [9] have been provided (Tables 3, 4). These criteria may be useful for the early diagnosis of MAS associated with other rheumatic diseases such as dermatomyositis [10]. In the present case, we suspected MAS at an early stage based on the patient's clinical symptoms and laboratory findings. The patient met the early diagnostic criteria for sJIA-MAS and SLE-MAS, and we diagnosed her with MAS and initiated treatment before the underlying disease was identified. Since the underlying condition is often unknown at the onset of secondary HLH, it may be necessary to initiate treatment based on these criteria when HLH is suspected.

**Table 2:** Diagnostic guidelines for HLH revised by the Histiocyte Society in 2004 Comments: (A) If hemophagocytic activity is not proven at the time of presentation, further search for hemophagocytic activity is encouraged. If the bone marrow specimen is not conclusive, material may be obtained from other organs. Serial marrow aspirates over time may also be helpful. (B) The following findings may provide strong supportive evidence for the diagnosis: (a) spinal fluid pleocytosis (mononuclear cells) and/or elevated spinal fluid protein, (b) histological picture in the liver resembling chronic persistent hepatitis (biopsy). (C) Other abnormal clinical and laboratory findings consistent with the diagnosis are: cerebromeningeal symptoms, lymph node enlargement, jaundice, edema, and skin rash. Hepatic enzyme abnormalities, hypoproteinemia, hyponatremia, increased VLDL, decreased HDL. *: hemoglobin <10 g/dL (in infants <4 weeks )

The diagnosis of hemophagocytic lymphohistiocytosis (HLH) can be established if one of either (1) or (2) below is fulfilled.	Our patient
(1) A molecular diagnosis consistent with HLH	
(2) Diagnostic criteria for HLH fulfilled (five out of the eight criteria below)	
Fever	+
Splenomegaly	
Cytopenias (affecting ≥2 of 3 lineages in the peripheral blood): Hemoglobin <9.0 g/dL*, Platelets <100×10^3^/μL, Neutrophils <1.0×10^3^/μL	
Hypertriglyceridemia and/or hypofibrinogenemia: Fasting triglycerides ≥3.0 mmol/L (i.e., ≥265 mg/dL), Fibrinogen ≤150 mg/dL	
Hemophagocytosis in bone marrow, spleen, or lymph nodes. No evidence of malignancy	
Low or absent NK-cell activity (according to local laboratory reference)	
Ferritin ≥500 ng/mL	+
Soluble CD25 (i.e., soluble interleukin-2 receptor) ≥2,400 U/ml	

**Table 3:** Criteria for the classification of macrophage activation syndrome in patients with systemic juvenile idiopathic arthritis Laboratory abnormalities should not be otherwise explained by the patient’s condition such as concomitant immune-mediated thrombocytopenia, infectious hepatitis, visceral leishmaniasis, or familial hyperlipidemia. *: Our patient did not meet this criterion on day 4 at the onset of MAS, but met it on day 5 with a triglycerides level of 172 mg/dL.

A febrile patient with known or suspected systemic juvenile idiopathic arthritis is classified as having macrophage activation syndrome (MAS) if both (1) and (2) below are met.	Our patient
(1) Ferritin >684 ng/mL	+
(2) Any two of the following:	+
Platelet count ≤181×10^3^/μL	+
Aspartate aminotransferase >48 U/L	+
Triglycerides >156 mg/dL	+^*^
Fibrinogen ≤360 mg/dL	+

**Table 4:** Preliminary diagnostic guidelines for macrophage activation syndrome as a complication of juvenile systemic lupus erythematosus Bone marrow aspiration for evidence of macrophage hemophagocytosis may be required only in doubtful cases. These criteria were developed using patients with active juvenile systemic lupus erythematosus (SLE) without MAS as a control group. As such, they may not be powerful enough to distinguish MAS from particular infectious complications. *: Our patient did not meet this criterion on day 4 at the onset of MAS, but met it on day 6 with a triglycerides level of 208 mg/dL.

The diagnosis of macrophage activation syndrome (MAS) requires the simultaneous presence of at least 1 clinical criterion and at least 2 laboratory criteria	Our patient
(1) Clinical criteria	
Fever (>38 ℃)	+
Hepatomegaly (≥3 cm below the costal arch)	
Splenomegaly (≥3 cm below the costal arch)	
Hemorrhagic manifestations (purpura, easy bruising, or mucosal bleeding)	
Central nervous system dysfunction (irritability, disorientation, lethargy, headache, seizures, or coma)	+
(2) Laboratory criteria	
Cytopenia affecting 2 or more cell lineages (white blood cell count ≤4000/μL, hemoglobin ≤9.0 g/dL, or platelet count ≤150×10^3^/μL)	+
Increased aspartate aminotransferase (>40 U/L)	+
Increased lactate dehydrogenase (>567 U/L)	+
Hypofibrinogenemia (fibrinogen ≤150 mg/dL)	
Hypertriglyceridemia (triglycerides >178 mg/dL)	+*
Hyperferritinemia (ferritin >500 ng/mL)	+
(3) Histopathologic criterion	
Evidence of macrophage hemophagocytosis in the bone marrow aspirate	

Monitoring a patient’s condition using cytokine profiles is clinically valuable in understanding the pathogenesis of MAS [11]. Primary MAS can cause a cytokine storm due to genetic abnormalities that disrupt the mechanisms controlling the abnormal activation of cytotoxic T and NK cells, whereas the mechanisms of secondary MAS remain unclear [1]. Some genetic and environmental factors are believed to stimulate macrophages to produce various inflammatory cytokines, leading to abnormal immune activation and subsequent cytokine storms. The disease activity of MAS is reflected differently by distinct inflammatory cytokines, depending on the underlying disease. Serum interleukin (IL)-18 levels may be associated with disease activity in patients with sJIA-MAS [12], whereas tumor necrosis factor (TNF)-α levels may be related to disease activity in those with SLE-MAS [13]. In our case, serum cytokine profiling in the acute phase revealed an overall significant increase in inflammatory cytokine levels, including IL-6 (63 pg/mL), IL-18 (1,145 pg/mL), C-X-C motif chemokine ligand 9 (CXCL9; 14,502 pg/mL), soluble tumor necrosis factor-receptor II (sTNF-RII; 13,893 pg/mL), and interferon (IFN)-α (27 pg/mL). CXCL9 is a cytokine that induces IFN-γ production while sTNF-RII is elevated along with TNF-α, reflecting its activity. According to the report by Mizuta et al. [11], serum IL-6 levels in patients with sJIA-MAS vs. active-phase sJIA, and SLE-MAS vs. active-phase SLE were 15 (3-352) pg/mL vs. 39.5 (3-870) pg/mL, and 6 (3-242) pg/mL vs. 4.5 (3-90) pg/mL, respectively, serum IL-18 levels were 172,000 (30,500-830,000) pg/mL vs. 31,150 (2,510-340,000) pg/mL, and 1,640 (430-14,100) pg/mL vs. 825 (225-1,410) pg/mL, respectively, serum sTNF-RII levels were 22,500 (8,600-66,300) pg/mL vs. 5,850 (1,060-34,300) pg/mL, and 32,000 (9,750-58,300) pg/mL vs. 9,450 (5,300-18,000) pg/mL, respectively. Serum ferritin levels, which are clinically used as indicators of disease activity in MAS, were correlated with serum sTNF-RII levels in patients with sJIA. Compared with these data, our patient exhibited relatively high levels of IL-6 and sTNF-RII but only slightly elevated levels of IL-18. Given that MCTD-MAS is expected to show a cytokine profile similar to that of SLE-MAS, the present case’s cytokine profile suggested an early stage of MAS associated with rheumatic diseases, including SLE and MCTD, rather than sJIA-MAS. We confirmed that the levels of these cytokines and ferritin had improved at a follow-up visit after the completion of treatment with steroid pulse therapy (Table 1).

Owing to overlapping clinical features and laboratory findings, differentiating MCTD from SLE in pediatric patients is challenging. In our case, she experienced transient headaches, altered mental status, and monoarthritis; however, the skin lesions characteristic of SLE, polyarthritis, photosensitivity, oral ulceration, or serositis were not observed. Laboratory test results showed leukopenia, positive antinuclear antibodies of anti-U1-RNP antibodies, and weakly positive anti-Sm antibodies; however, decreases in the levels of other blood cells and complement activity and anti-dsDNA and anti-phospholipid antibodies were not detected. No significant proteinuria was observed, and her renal histology was normal. In Japan, the diagnosis of childhood SLE is based on the Guideline for the Diagnosis of SLE in Child [14], which is based on the American College of Rheumatology’s classification criteria in 1997 (ACR-1997 criteria) [15]. Globally, the Systemic Lupus International Collaborating Clinics classification criteria in 2012 (SLICC-2012 criteria) [16] and SLE classification by European League Against Rheumatism/American College of Rheumatology in 2019 (EULAR/ACR-2019 criteria) [17] have been developed. Our case did not meet any diagnostic criteria except for the EULAR/ACR-2019 criteria. However, these criteria are adult-oriented and place significant emphasis on anti-Sm antibody positivity, which was only weakly positive in our patient's blood tests. Moreover, a systematic review and network meta-analysis by Chang et al. reported that the EULAR/ACR-2019 criteria had a lower sensitivity than the SLICC-2012 criteria, and the specificity was lower than that of the ACR-1997 criteria in patients with pediatric SLE [18]. Thus, the EULAR/ACR-2019 criteria may not frequently be suitable for cases of pediatric SLE. Conversely, our case met the Japanese diagnostic criteria for MCTD in pediatric patients. Our patient exhibited Raynaud’s phenomenon, strong positivity for anti-U1-RNP antibodies, and leukopenia, resulting in the diagnosis of MCTD with the criteria proposed by Tanaka et al. in 2019 [19]; however, she did not meet the criteria established by Alarcón-Segovia in 1987 [20]. Therefore, we determined that MCTD was a more appropriate diagnosis than SLE, based on the pediatric criteria used in our country. To date, her condition has been well-managed with low-dose oral prednisolone; however, she requires close monitoring for the potential development of scleroderma- and polymyositis-like manifestations.

## Conclusions

MAS is a life-threatening complication of rheumatic diseases. We experienced a rare case of MAS as an atypical manifestation of MCTD in a 14-year-old adolescent girl. Early diagnosis and successful treatment were achieved using the early diagnostic criteria for MAS associated with other rheumatic diseases. This case highlights the importance of early diagnosis and prompt treatment, even when the underlying disease is initially unknown. Additionally, cytokine profiling may provide valuable insights into understanding the pathogenesis of diseases that cause hypercytokinemia.
